# Circadian Cycle-Dependent MeCP2 and Brain Chromatin Changes

**DOI:** 10.1371/journal.pone.0123693

**Published:** 2015-04-13

**Authors:** Alexia Martínez de Paz, Jose Vicente Sanchez-Mut, Mireia Samitier-Martí, Paolo Petazzi, Mauricio Sáez, Karolina Szczesna, Dori Huertas, Manel Esteller, Juan Ausió

**Affiliations:** 1 Cancer Epigenetics and Biology Program (PEBC), Bellvitge Biomedical Research Institute (IDIBELL), Barcelona, Catalonia, Spain; 2 Department of Physiological Sciences II, School of Medicine, University of Barcelona, Barcelona, Catalonia, Spain; 3 Institució Catalana de Recerca i Estudis Avançats (ICREA), Barcelona, Catalonia, Spain; 4 Department of Biochemistry and Microbiology, University of Victoria, Victoria, British Columbia, Canada; University of Insubria, ITALY

## Abstract

Methyl CpG binding protein 2 (MeCP2) is a chromosomal protein of the brain, very abundant especially in neurons, where it plays an important role in the regulation of gene expression. Hence it has the potential to be affected by the mammalian circadian cycle. We performed expression analyses of mice brain frontal cortices obtained at different time points and we found that the levels of MeCP2 are altered circadianly, affecting overall organization of brain chromatin and resulting in a circadian-dependent regulation of well-stablished MeCP2 target genes. Furthermore, this data suggests that alterations of MeCP2 can be responsible for the sleeping disorders arising from pathological stages, such as in autism and Rett syndrome.

## Introduction

Many physiological aspects of a wide range of organisms, from cyanobacteria to mammals, display circadian oscillations as a mechanism to adapt to the 24 hours light-dark cycles [1–3].

In mammals, the central biological clock is found in the suprachiasmatic nucleus (SCN) of the hypothalamus, light being the main environmental stimulus capable of resetting the clock. The retina receives the light signals and sends this information through the retinohypothalamic tract to the SCN, which in turn synchronizes peripheral clocks present in most organs [2], reviewed in [4].

At the molecular level, the circadian cycle is regulated by positive and negative feedback mechanisms [5, 6]. In mammals, the BMAL1 (Brain and muscle Arnt-like protein 1) and CLOCK (Circadian locomotor output cycles kaput) proteins heterodimerize and act as transcriptional activators of the *Cry* (Cryptochrome) and *Per* (Period) genes. When the oligomeric CRY and PER proteins associate, they enter the nucleus and inhibit BMAL1 and CLOCK transcriptional activity, thus repressing their own transcription [2, 7].

An extra layer of epigenetic regulation adds to the traditional view of the molecular clock as a result of transcriptional-translational feedback loops. Changes in chromatin structure are necessary to allow or prevent expression of genes. Therefore, it is not surprising that the epigenetic machinery plays an important role in circadian phase-shifting. Numerous studies have demonstrated the recruitment of epigenetic proteins and rhythmic histone modifications at circadian gene promoters driving cyclic expression [8–11]. Indeed, CLOCK itself has histone acetyl transferase activity [12], and PER proteins interact with several histone de-acetyl transferases and methyltransferases [13]. Furthermore recent findings reported genome-wide 24-hour rhythms of DNA methylation, specially, near transcription start sites [14].

MeCP2 is a chromatin bound protein [15] that is very abundant in the brain. Particularly, it is expressed at similar levels of those of H1 in neurons [16, 17], pointing towards a major function in chromatin regulation in these cells. MeCP2 was initially described as a global transcriptional repressor [18], although a more complex behaviour has been recently identified, showing that MeCP2 can act either as an activator or as a repressor, depending on genes and the co-factors with which it is associated [19]. Mutations in MeCP2 cause Rett syndrome [20], which is frequently associated with circadian disturbances and sleep-wake disorders [21]. Previous data supports these observations, and suggest that MeCP2 could be involved in the circadian rhythm regulation [22].

Herein, we deepen in this possibility by demonstrating that not only is MeCP2 circadianly regulated, but also, that it has important consequences on the chromatin structure and transcription of MeCP2 dependent genes.

## Materials and Methods

### Animals

All animals used in this work were derived from C57BL/6 mice maintained under standard animal house conditions (12 hour dark-light cycles on *ad libitum* food and water intake). Three months mice were euthanized every 6 hours from first light *stimulus* on (Lights “ON” are designated as Zeitgeber time [ZT] 0) by exposure to CO_2_ and brains were rapidly removed, dissected, and the different brain areas kept at -80°C until further use. The experimental procedures were in agreement with all legislation defined by the European Union and approved by the local ethics committee (UB-IDIBELL).

### Chromatin fractionation analysis

Nuclei from frontal cortices were isolated and diluted in micrococcal nuclease digestion buffer (50 mM NaCl, 10 mM PIPES pH 6.8, 5 mM MgCl_2_ and 1 mM MgCl_2_). Nuclei were pre-warmed at 37°C and MNase (Roche) digested (2 U/mg tissue). Reactions were stopped on ice with 5 mM EDTA). Before fractionation, 5% of total volume was separated to use it as input DNA. Afterwards nuclei were centrifuged at 8000 g for 10 minutes, and supernatant (euchromatin-enriched phase) was obtained. Pellets were re-suspended in 0.25 mM EDTA, vortexed, incubated 1 hour at 4°C and centrifuged to obtain a supernatant which is substantially enriched in facultative heterochromatin domains [23]. The remaining phase corresponds to constitutive heterochromatin and the regions corresponding to transcriptionally active promoters that are loaded with the largely insoluble basal transcriptional machinery complexes. DNA from each of the aforementioned phases was purified by SDS-protein salt precipitation, and DNA concentration normalized using the Qubit 2.0 Fluorimeter and dsDNA Broad Range Assay reagents (Life Technologies, Carlsbad, CA, USA). Chromatin accessibility was analyzed by quantitative PCR of genes of interest and expressed as a ratio between euchromatin and heterochromatin (facultative + constitutive).

### Western blot analysis

Frozen frontal cortices were homogenized on Laemmli buffer (2% SDS, 10% glycerol, 0.002% bromophenol blue and 62.5 mM Tris-HCl pH 6.8). Protein content was measured by the Lowry method (Bio-Rad Hercules, CA, USA). Immediately after, 3% B-mercaptoethanol was added; proteins were separated by sodium dodecyl sulfate polyacrylamide gel electrophoresis (SDS-PAGE), and transferred onto nitrocellulose membranes (GE Healthcare, Pittsburgh, PA, USA). Nonspecific bindings were blocked by incubation in 5% skimmed milk in phosphate buffered-saline pH 7.2 with 0.1% Tween 20. Membranes were incubated with specific antibodies, either overnight at 4°C or for 1 hour at room temperature. Antibodies and dilutions used were as follows: MeCP2 (Sigma-Aldrich, St Louis, MO, USA) 1:5000, Clock (Cell Signaling, Beverly, MA, USA) 1:1000 and β-actin (Sigma-Aldrich, St Louis, MO, USA) 1:20000, horseradish peroxidase-conjugated (HRP) secondary antibodies (GE Healthcare) 1:10000. Densitometric analyses were performed with Fiji software [24].

### RNA extraction and reverse-transcription

Total RNA purification was performed by homogenizing frozen tissue in Ribozol (Amresco, Solon, OH, USA) following manufacturer instructions. RNA was reverse-transcribed using the Thermoscript RT-PCR system (Invitrogen, Carlsbad, CA, USA).

### Real-time polymerase chain reaction

Each PCR was carried out in triplicate using SYBR Green PCR Master Mix (Applied Biosystems-Life Technologies) and 5 ng cDNA, or 2 ng in case of genomic DNA. PCR conditions were 10 minutes at 95°C, then 40 cycles of 15 seconds at 95°C and 1 minute at 60°C. Fluorescent signals were acquired by the ABI Prism 7900HT Sequence Detection System (Applied Biosystems), and positive standard deviations were normalized using three housekeeping genes (*PPIA*, *RPL38* and *TBP*). PCR efficiencies were calculated using standard dilutions and LinReg software [25]. The primers used were as follows; *Clock* (fwd: GAGGTCGTCCTTCAGCAGTC, rv: CGCTGCTCTAGCTGGTCTTT), *Mecp2* (fwd: GCTTCTGTAGACCAGCTCCAA, rv: ATAATGGAGCGCCGCTGTTT), *Bdnf* (fwd: CTCCGCCATGCAATTTCCAC, rv: GCCTTCATGCAACCGAAGTA), *Sst* (fwd: AACAGGAACTGGCCAAGTACT, rv: GGGCTCCAGGGCATCATTCTC), *Dlk1* (fwd: TCGGCAGCCGCACTTAGCAG, rv: GGTTGCGGACCACGAAGGGG), *Ppia* (fwd: CAAATGCTGGACCAAACACAAACG, rv: GTTCATGCCTTCTTTCACCTTCCC), *Rpl38* (fwd: AGGATGCCAAGTCTGTCAAGA, rv: TCCTTGTTGTGATAACCAGGG), *Tbp* (fwd: CCCCACAACTCTTCCATTCT, rv: GCAGGAGTGATAGGGGTCAT), genomic_*Bdnf* (fwd: TGATCATCACTCACGACCACG, rv: CAGCCTCTCTGAGCCAGTTACG), genomic_*Sst* (fwd: ATTTTGCGAGGCTAATGGTG, rv: TATGGAGCTCTCCACGGTCT), genomic_*Dlk1* (fwd: GCTTGGTTCCTGAGACTTGC, rv: CGCACTCTTTGGCTAGACG).

## Results

### MeCP2 displays circadian oscillations

**Fig 1:**
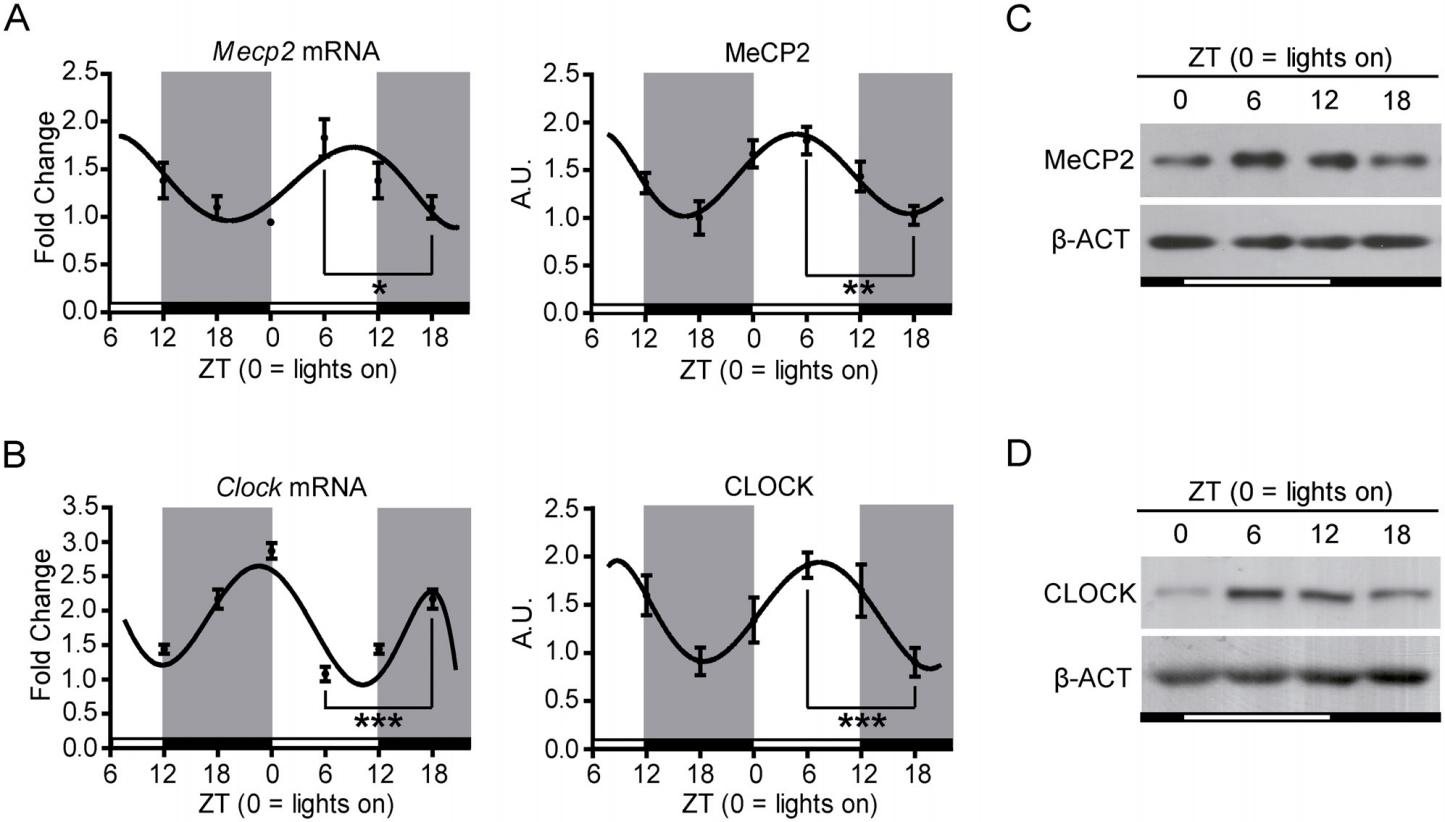
Circadian oscillations of MeCP2 and CLOCK proteins in mouse *frontal cortex*. RT-qPCR results and densitometric analysis of WB experiments, showing differences in **(A)** MeCP2 and **(B)** CLOCK expression. Mice were under constant 12 hour light-dark cycles (white and grey background represent lights on and off, respectively) and were processed at the indicated ZTs (Zeitgeber times in hours) (n = 10/ time point, means ± SEM are represented). Both MeCP2 and CLOCK levels are significantly different between ZTs 6 and 18. **(C), (D)** Representative MeCP2 and CLOCK WBs (white and black bars represent, respectively, lights on and off). *P<0.05, **P<0.005, ***p<0.0005 in two-tailed Student’s *t*-tests.

As we have mentioned, MeCP2 is an important player in neuronal chromatin structure [15–17]. It has recently been suggested that MeCP2 might be influenced by circadian rhythms [22] but, in spite of the importance of this aspect, the circadian regulation of MeCP2 has been poorly investigated. To study this further, we decided to use C57BL6 wild type mice and to focus on a specific brain region, the frontal cortex, because it is especially relevant for MeCP2 function [26–28]. MeCP2 ablation in forebrain neurons is sufficient to induce several Rett-like behavioral impairments [26, 28], and MeCP2 levels in frontal cortex are particularly correlated with the overall phenotypic severity of mice models of Rett syndrome [28]. Of note, frontal cortex MeCP2 levels also correlate with the abnormal 24-hour electroencephalogram (EEGs) activity observed in Rett syndrome [28] supporting the role of MeCP2 in sleep-wake regulation. Four zeitgeber times (ZT0, ZT6, ZT12 and ZT18) and 10 animals per time-point were considered. As a result, 40 samples were analyzed. The results thus obtained unambiguously showed circadian oscillations of the MeCP2 RNA and protein levels (P-val<0.1 and P-val<0.01 respectively; Fig 1A) that resemble those observed for the well-known circadian gene CLOCK (Fig 1B). The highest and lowest levels of MeCP2 were observed at ZT 6 and 18 (Fig 1A and 1C), corresponding to the sleep and wake phases of the circadian cycle respectively. By contrast, ZT 0 and 12 showed intermediate levels, suggesting that they correspond to transition stages between the ZT 6 and 18 phases. These data are in sharp contrast with previous observations suggesting that MeCP2 protein levels are constant during 9 hours in wild-type cortical neurons [29]. This discrepancy prompted us to further investigate the MeCP2 half-life by blocking *de novo* protein synthesis using cycloheximide (CHX) in BJ fibroblasts overexpressing MeCP2 protein. In line with the circadian regulation of MeCP2, a fast decrease of MeCP2 levels was observed within the first 6 hours, with a half-life of life of approximately 5.2 hours (S1 Fig), which is in strong agreement with the observed 12 h-period MeCP2 oscillations.

### MeCP2 oscillations result in global chromatin alterations

Once the circadian regulation of MeCP2 was established, we wondered how chromatin structure might be affected by MeCP2 oscillations. To address this issue, we investigated the MNase accessibility to promoter regions of three known MeCP2 target genes, brain derived neurotrophic factor *(Bdnf)*, delta-like 1 homolog *(Dlk1)*, and somatostatin *(Sst)* [19, 30]. Similarly, four ZT times and 3–5 samples per time were digested with micrococcal nuclease (MNase), and the chromatin fragments thus obtained were analyzed by quantitative PCR.

**Fig 2:**
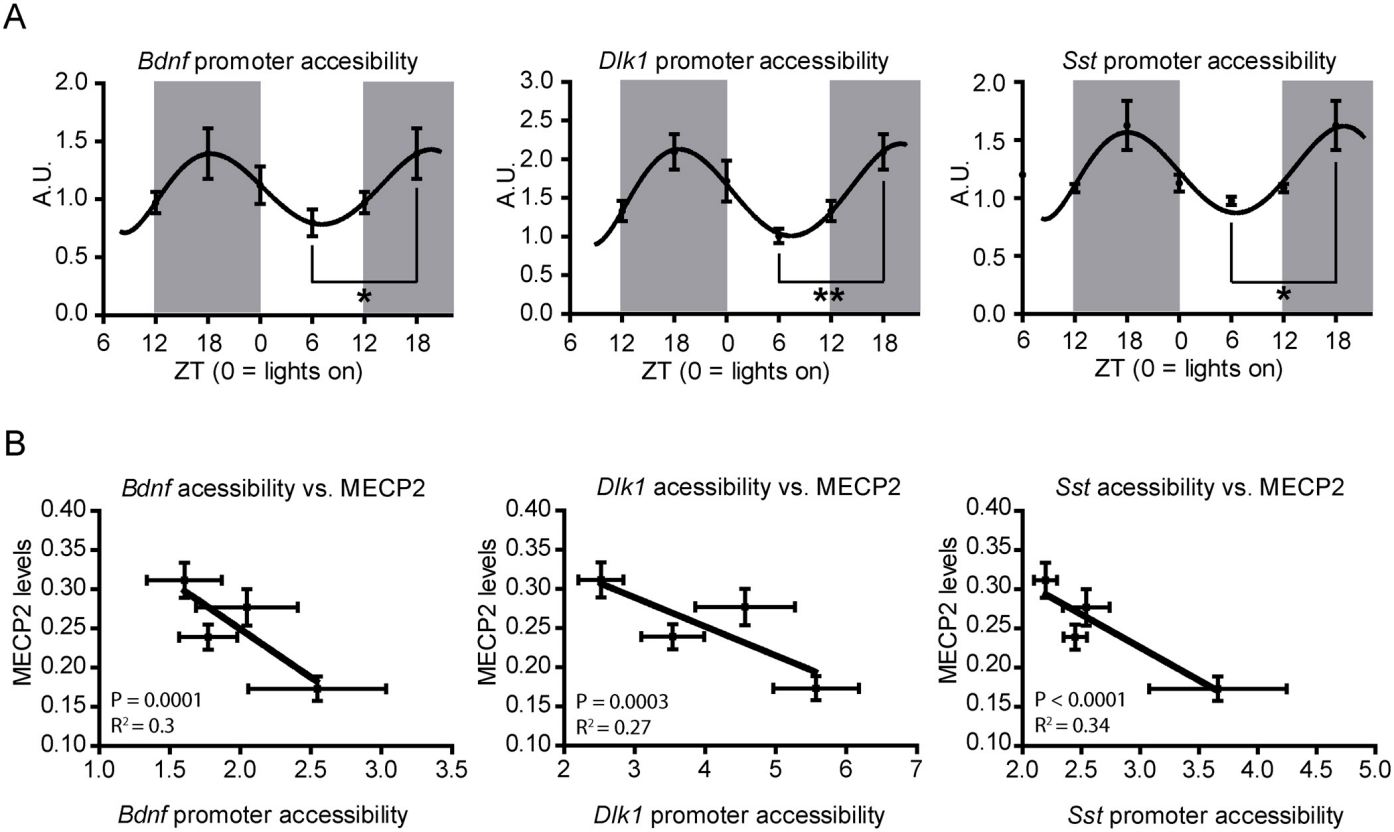
Changes in chromatin accessibility at the *Bdnf*, *Dlk* and *Sst* promoters correlate with MeCP2 levels. **(A)** MNase digestions, chromatin fractionation, and following RT-qPCRs revealed in all three cases a progression from a less accessible status at ZT 6 to a transcriptionally permissive status at ZT 18 (n = 3–5 mice/time point; graphs show mean ± SEM). **(B)** Correlation of promoter accessibility with MeCP2 levels. *P<0.05, **p<0.005 in two-tailed Student’s *t*-tests. Correlation p-values correspond to the linear regression coefficient.

In agreement with the circadian regulation of MeCP2, the MNase assay showed that promoters of MeCP2 target genes display differences in accessibility following a circadian pattern (P-val<0.05; Fig 2A). We found that ZT6 and ZT18 displayed the lowest and highest MNase accessibility levels, showing intermediate values ZT0 and ZT12 (Fig 2A) indicating a higher and lower promoter occupancy at ZT6 and ZT18, and intermediate levels at ZT0 and ZT12.

Interestingly, this pattern was inversely correlated with MeCP2 protein levels (P-val<0.001; Fig 2B), suggesting that higher levels of MeCP2 are associated with a less accessible DNA chromatin organization.

### Changes in circadian cycle-dependent chromatin landscape affect the expression of MeCP2-regulated genes

The results described in the previous section prompted us to investigate whether gene expression might also be affected. To this end, 3–5 samples for each of the four ZT were analyzed by quantitative PCR.

**Fig 3:**
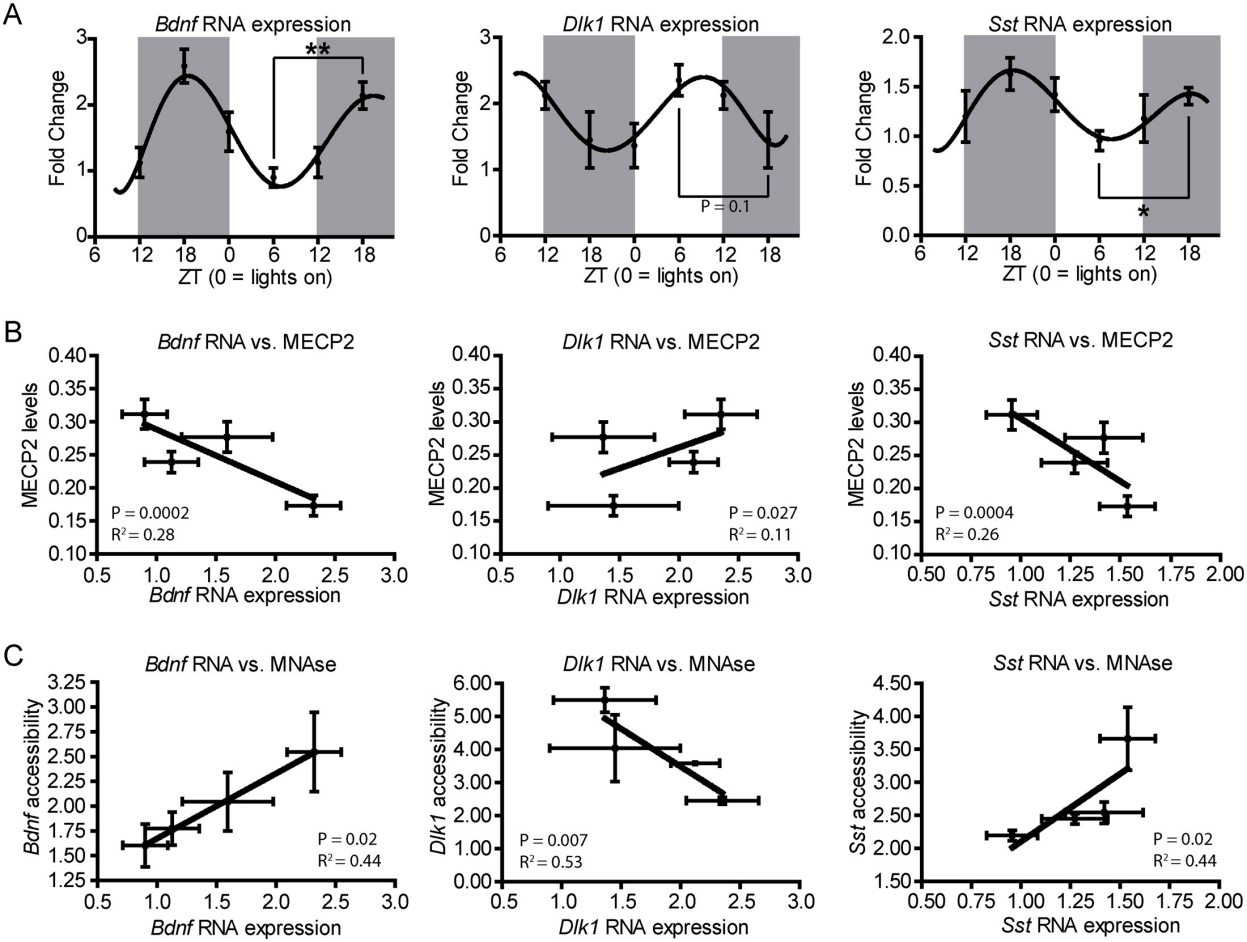
MeCP2-targets expression patterns also exhibit circadian changes. **(A)** RT qPCR analysis demonstrates gene expression changes along light-dark cycles (n = 3–5 mice/time point; graphs show mean ± SEM). **(B)** Correlations between MeCP2-targets expression with MeCP2 protein levels as well as **(C)** with the accessibility to the corresponding promoters. *P<0.05, **p<0.005 in two-tailed Student’s *t*-tests. Correlation p-values correspond to the linear regression coefficient.

The results showed that *Bdnf* as well as *Dlk1* and *Sst* displayed in all instances circadian patterns of expression (Fig 3A) in a similar way as the MeCP2 variation (Fig 1) and MNase accessibility levels (Fig 2). The highest differences were observed between ZT 6 and ZT 18, showing intermediate levels at ZT 0 and ZT 12 (Fig 3A). Curiously, RNA expression levels were positively and negatively correlated with MNase and MeCP2 protein levels respectively in *Bdnf* and *Sst* genes (P-val<0.001; Fig 3B and 3C), pointing towards a repressive role of MeCP2 in their regulation. On the contrary, the inverse relation was observed in the *Dlk*1 gene suggesting, in this case, a positive role of MeCP2 in the expression of this gene in the frontal cortex (P-val<0.05; Fig 3B and 3C). Supporting the involvement of MeCP2 in the circadian regulation of these genes, a dampened pattern of oscillations was observed in MeCP2 KO mice (S2 Fig).

## Discussion

Several lines of evidence point out the importance of epigenetic players in circadian regulation [8–13], and MeCP2 is not an exception. In line with previous observations [22], our data indicates that brain chromatin structure is subject to circadian cycle regulation in a process in which MeCP2 is involved. To our knowledge, this is the first report providing direct evidence of MeCP2 circadian regulation and its functional consequences. Furthermore, our findings suggest a direct correlation between the circadian regulation of MeCP2 protein levels and the accessibility and transcription of MeCP2 target genes. In the brain, this chromosomal protein is highly abundant: in neurons it is present in about one molecule for every two nucleosomes [16], and in the overall unfractionated brain it is present in one over three nucleosomes [17]. Such abundance implies a fairly ubiquitous distribution of MeCP2 along the brain chromatin [16, 31]. Although the exact details of such a genome-wide distribution are not yet clearly understood, any disturbances in its global content may, unsurprisingly, affect the expression of genes whose expression is directly regulated by this protein.

It is important to mention that the expression patterns of some of the MeCP2-regulated genes analyzed here do not completely correlate with their previously described repressive-activating function of MeCP2. In this regard, while our data is in line with the extensively documented repressive role of MeCP2 in *Bdnf* transcription [32–34] reviewed in [35], it disagrees with the limited prior information available on the expression of the *Dlk1* and *Sst* genes [19, 30]. *Dlk1* and *Sst* genes were found up and down regulated in MeCP2-KO mice [19, 30], pointing toward a repressive and activating role of MeCP2 respectively. In contrast, our data suggest that MeCP2 is positively and negatively correlated with the expression of *Dlk1* and *Sst*. Noticeably, our data refers to the circadian regulation and MeCP2 occupancy on gene promoter regions, which can be subjected to different mechanisms of regulation that the overall MeCP2 genomic occupancy. In fact, MeCP2 recognizes DNA methylation which exerts opposite effects in gene expression in promoter regions and gene bodies [36]. Also, it is possible that these discrepancies may be a consequence of different experimental approaches. In this regard, several matters need to be taken into consideration: First of all, it should be taken into account that the brain is a very heterogeneous system, in which every discrete region exhibits a different organization, cell content, and function [37, 38]. Therefore, the MeCP2 behaviour described for a given region should not necessarily be similar in other regions. Because of this, different analyses from different brain regions might produce contradictory results. Indeed, previous data have provided evidence for the existence of distinct MeCP2-target genes in the cerebellum and hypothalamus [39]. Second, a very important part of studies on MeCP2 function are based on MeCP2-KO and MeCP2-Tg mice models, where the protein is either lacking or its levels of expression are increased, respectively. While informative, data obtained from these systems should not necessarily be complementary or similar to those obtained with a more physiologically relevant system, such as the one used here. Third, it is noteworthy to point out the high levels of discrepancy between the different reports on the MeCP2-related gene expression changes, which do not always share the same directionality [19, 32, 39–41]. Finally—and significantly—the circadian dependence of some MeCP2-regulated genes, such as those analysed by us, indicate that a significant part of the expression discrepancies might be a consequence of the samples being analyzed during different windows of time. Therefore, any future studies aimed at unravelling the MeCP2 function should not only carefully consider specific cell type and brain region, but also consider the specific time frame at which the samples were collected. This should help provide a more comprehensive view, and also enhance the reproducibility of the results from different laboratories and studies.

**Fig 4:**
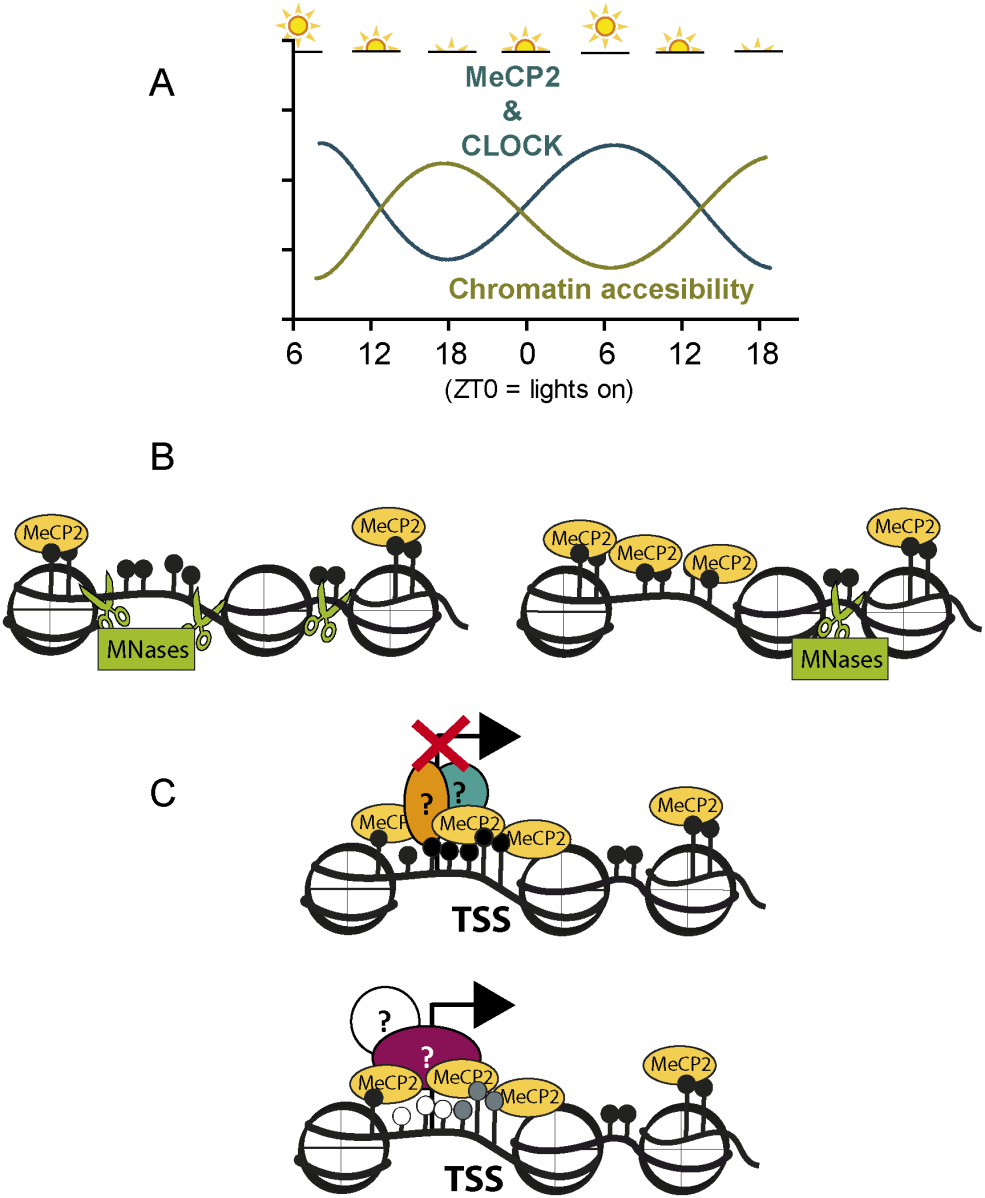
Cartoon representation of the transitions observed during the mouse circadian cycle. **(A)** The circadian variation of CLOCK and MeCP2 are accompanied by changes in the overall accessibility of brain chromatin to Mnase digestion. **(B)** Schematic representation of the MeCP2 changes observed during the circadian cycle. The overall increase in the MeCP2 level results in changes in its binding to the chromatin substrate, resulting in global changes in micrococcal nuclease accessibility (green scissors). **(C)** Additional MeCP2 binding to 5-hydroxymethylcytosines or 5-methylcytosines of certain chromatin domains and its possible association with different protein partners, results in the enhancement of its dual transcriptional activity, leading to either activation or repression of gene expression. In this representation, nucleosomes are in white, DNA is indicated as a black line, CpG dinucleotides are represented as lollipop structures black colored when methylated, grey when hydroximethylated and white when unmodified; MeCP2 partners are depicted as ovals or circles with question marks.

Altogether, the data provided in Figs 1, 2, 3 suggest that brain chromatin is subject to circadian cycle regulation in a process involving MeCP2. According to our data, we propose a model (Fig 4) in which, through the circadian regulation of MeCP2, DNA accessibility and gene expression are circadian cycle regulated. In this model, the increase in the MeCP2 levels observed during the sleeping phase (Fig 4A) induces an increase in the overall genomic MeCP2 occupancy and the corresponding decrease in MNase accessibility (Fig 4B). These global MeCP2 and chromatin changes cause circadian cycle-dependent alterations in MeCP2-regulated genes, showing different states at sleep and wake phases, surrounded by two intermediate states at ZT 0 and ZT 12 (*i*.*e*. a decrease in the expression of *Bdnf* and *Sst* genes and an increase in the expression of *Dlk1* which are correlated with the increase in MeCP2 during the sleeping phase of the circadian transition). It is interesting to note that, conversely, mis-timed sleep has been shown to disrupt the circadian regulation of the human transcriptome [42].

The transcriptional changes associated with the circadian-cycle dependent MeCP2 oscillations in the genes—described here—are very relevant to normal brain physiology. *Bdnf* is involved in cognitive functions [43] which are modulated by circadian rhythms [44]. Somastotatin signaling pathways are very important for the adaptive stress response [45] and have been shown to play an important role in the circadian modulation of anxiety [46]. *Dlk1* is an important player in the determination of fast motor neurons [47]. All in all, the MeCP2 oscillations described here may play an important role in the sleeping disturbances that, in autistic diseases including Rett syndrome, involve alterations of the MeCP2 function [48, 49] and appear to also be frequent in other neurologic diseases such as Parkinson’s, Alzheimer’s, and schizophrenia or anxiety-related disorders [50].

## Supporting Information

S1 Fig MeCP2 half-life is in agreement with MeCP2 circadian oscillations. MeCP2 half-life assayed in BJ cells (human normal fibroblasts) transduced with a pLVX-IRES-zsGreen-MeCP2 construct. **(A)** Western-blot of cells treated with 75 μg/mL cycloheximide de novo protein synthesis blocking reagent at different times. **(B)** Representation of the raw data of MeCP2 levels normalized with actin. **(C)** MeCP2 half-life (t_½_) determined according to the linear regression of logarithmic transformed MeCP2 values and time.
(PDF)

S2 Fig MeCP2 absence impairs MeCP2-target genes circadian expression. MeCP2-target genes expression in wild-type and *MeCP2-*KO mice. 3–5 samples per group/time point were analyzed and mean ± SEM represented as a fold change in relation with the lowest value of the group. **P<0.005, ***P<0.0005 in two-tailed Student’s t-tests.
(PDF)
