# Correction: Circadian Cycle-Dependent MeCP2 and Brain Chromatin Changes

**DOI:** 10.1371/journal.pone.0136470

**Published:** 2015-08-20

**Authors:** The PLOS ONE Staff

## Notice of Republication

This article was republished on July 31, 2015, to correct the second author’s name in the citation. The publisher apologizes for the error. The correct citation is: Martínez de Paz A, Sanchez-Mut JV, Samitier-Martí M, Petazzi P, Sáez M, Szczesna K, et al. (2015) Circadian Cycle-Dependent MeCP2 and Brain Chromatin Changes. PLoS ONE 10(4): e0123693. doi:10.1371/journal.pone.0123693.

Please download this article again to view the correct version. The originally published, uncorrected article and the republished, corrected article are provided here for reference.

## Supporting Information

S1 File Originally published, uncorrected article. (PDF)

S2 File Republished, corrected article. (PDF)
