# Supplementary figures and images for: Circadian Cycle-Dependent MeCP2 and Brain Chromatin Changes
Source: PLoS One. 2015 Apr 13;10(4):e0123693. doi: 10.1371/journal.pone.0123693 (PMC4395115; doi:10.1371/journal.pone.0123693)

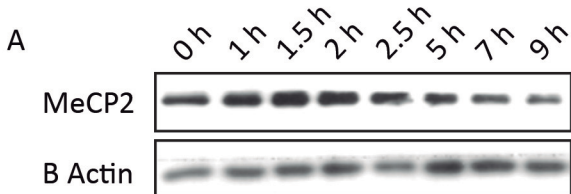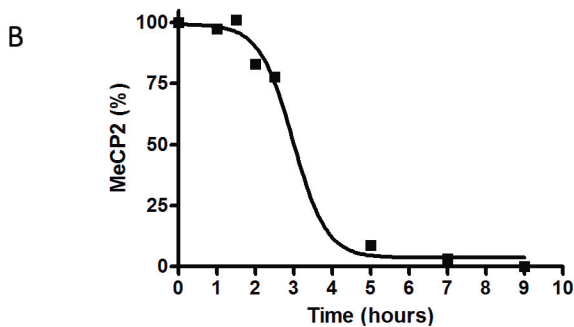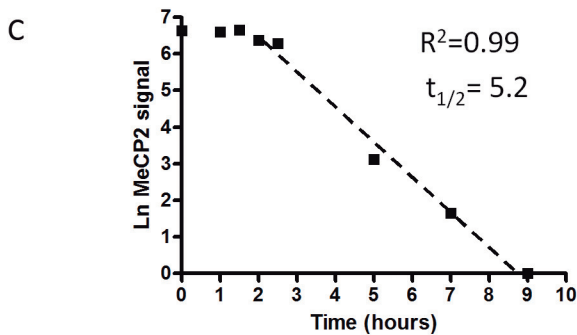

Supplement: S1 Fig — MeCP2 half-life assayed in BJ cells (human normal fibroblasts) transduced with a pLVX-IRES-zsGreen-MeCP2 construct. (A) Western-blot of cells treated with 75 μg/mL cycloheximide de novo protein synthesis blocking reagent at different times. (B) Representation of the raw data of MeCP2 levels normalized with actin. (C) MeCP2 half-life (t½) determined according to the linear regression of logarithmic transformed MeCP2 values and time. (PDF) [file pone.0123693.s001.pdf]

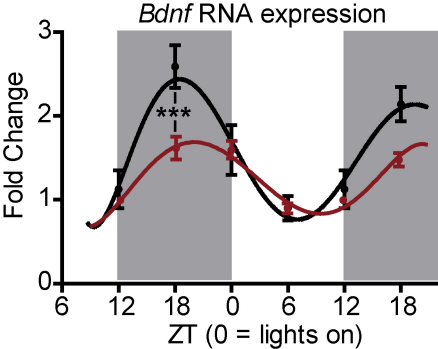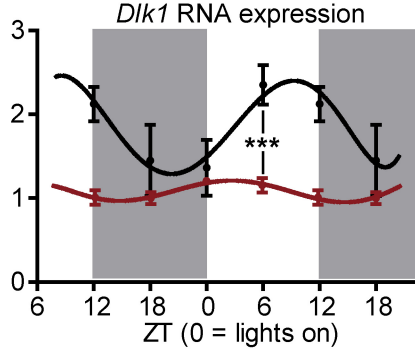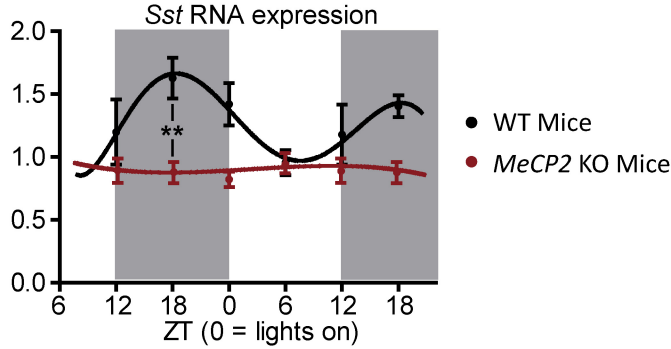

Supplement: S2 Fig — MeCP2-target genes expression in wild-type and MeCP2-KO mice. 3–5 samples per group/time point were analyzed and mean ± SEM represented as a fold change in relation with the lowest value of the group. **P<0.005, ***P<0.0005 in two-tailed Student’s t-tests. (PDF) [file pone.0123693.s002.pdf]
